# Pamidronate for Hypercalcemia in Critically Ill Surgical Patients

**DOI:** 10.7759/cureus.72922

**Published:** 2024-11-03

**Authors:** Lisa Hall Zimmerman, Heather S Dolman, Lauren Riley Howell, Janie Faris, William B Zimmerman, Alfred E Baylor, James Tyburski, Robert F Wilson

**Affiliations:** 1 Department of Pharmaceutical Services, William Beaumont University Hospital, Royal Oak, USA; 2 The Michael and Marian Ilitch Department of Surgery, Wayne State University School of Medicine, Detroit, USA; 3 Department of Surgery, Detroit Receiving Hospital, Detroit, USA; 4 Department of Pharmacy Services, Detroit Receiving Hospital, Detroit, USA; 5 Department of Osteopathic Surgical Specialties, Michigan State University, East Lansing, USA

**Keywords:** critically ill patients, hypercalcemia, pamidronate, severe hypercalcemia, surgical patient

## Abstract

Introduction: Hypercalcemia in critically ill patients is associated with an increased severity of illness and mortality that becomes worse as the levels rise. Pamidronate was evaluated for the treatment of hypercalcemia in critically ill surgical patients.

Methods: This retrospective study evaluated 30 critically ill surgical patients who developed hypercalcemia (ionized calcium (iCa)≥1.25 mmol/L) while in the surgical ICUs over three years. Patients were case-matched 1:1 for age and severity of illness.

Results: In the 30 patients with an overall Acute Physiology and Chronic Health Evaluation (APACHE) II of 22±9, mechanical ventilation was required in 27/30 (90%) and hemodialysis in 11/30 (37%). Within four days of pamidronate, iCa declined from a mean of 1.46±0.14 to 1.15±0.14 mmol/L, p=0.004; however, the blood urea nitrogen (BUN) and serum creatinine (SCr) increased significantly in patients with renal impairment. An iCa≥1.35 mmol/L increased mortality from 0% to 26%.

Conclusions: Hypercalcemia in critically ill surgical patients is associated with increased severity of illness. Over a period of four days, pamidronate reduced iCa levels at ~0.08 mmol/L/day and corrected 80% of the hypercalcemic patients; however, it may cause further renal dysfunction.

## Introduction

Hypercalcemia is relatively uncommon in critically ill patients but can occur in up to 23% of severely ill intensive care unit (ICU) patients [1,2]. Blood calcium levels are regulated primarily by parathyroid hormone (PTH) and vitamin D but may be altered by cytokines liberated by a variety of inflammatory responses [3,4]. As a result, an increase in resorption of calcium can occur from bones and the kidneys. In addition, prolonged immobilization can also increase the release of calcium from bones [5,6].

Hypercalcemia can have life-threatening effects, especially fatal arrhythmias, and muscle weakness [4,7-10]. Other side effects include polyuria resulting in dehydration and impaired renal function [3,9,10]. In a few case studies, administration of a bisphosphonate has been reported to reduce calcium resorption from bone which limits the severity of the hypercalcemia and the resultant negative sequelae [5-7]. The limited portions of the data from this manuscript were presented as an abstract at the Society of Critical Care Medicine Annual Congress, San Juan, Puerto Rico in January 2013. The objective of this study was to evaluate the effects of pamidronate on critically ill patients with hypercalcemia compared to the 15 other hypercalcemic patients who did not receive pamidronate.

## Materials and methods

Study population

This IRB-approved (Wayne State University, 076412M1E), retrospective, case-matched study was conducted in the surgical intensive care unit (ICU) and burn ICU in a university-affiliated, Level 1 Trauma hospital. The IRB granted exemption as this is a retrospective study. Patients were evaluated in a step-wise fashion to determine study eligibility and case-matched on a 1:1 basis using Acute Physiology and Chronic Health Evaluation (APACHE) II score, age, and admitting service. Groups were compared using pamidronate therapy versus control patients. Hospital and ICU records were assessed over three consecutive years. Patients were in the case and control groups with the following criteria: at least 18 years of age, required ICU admission for at least 48 hours and had at least one episode of hypercalcemia defined as serum ionized calcium of 1.25 mmol/L [1]. Patients were excluded in the case and control group for current pregnancy or a diagnosis of primary hyperparathyroidism. Criteria for the use of pamidronate were based on an episode of hypercalcemia and prescribed at the discretion of the primary team.

Hypercalcemia

Hypercalcemia was defined as a serum ionized calcium (iCa) of ≥ 1.25 mmol/L. Effectiveness was evaluated through the assessment of iCa and various laboratory studies. The resolution of hypercalcemia was two sequential iCa levels < 1.25 mmol/L. Conventional therapies evaluated for hypercalcemia included loop diuretics and intravenous fluids, primarily normal saline. Hypercalcemia was further classified by severity into mild (iCa 1.25-1.34 mmol/L), moderate (iCa 1.35-1.44 mmol/L), and severe (iCa ≥ 1.45 mmol/L).

Assessment of organ dysfunction

Safety was evaluated through the assessment of organ dysfunction. Bradycardia (heart rate < 60 beats per minute (bpm)), tachycardia (heart rate > 100 bpm), and other arrhythmias were identified from ICU vital signs flowsheets. Electrocardiogram (ECG) rhythm strips were evaluated at least twice daily. Moderate to severe renal dysfunction was defined as blood urea nitrogen (BUN) ≥ 40 mg/dL or SCr ≥ 2 mg/dL. The need for hemodialysis indicated severe renal dysfunction.

Statistical analysis

Statistical analysis was performed with IBM SPSS Statistics for Windows, version 21 (IBM Corp., Armonk, NY). Pearson's chi-square analysis was utilized for nominal data. Continuous data was evaluated using the student t-test for parametric data and the McNemar test for non-parametric data. No power analysis was conducted a prioriwith this retrospective review. A p-value of ≤ 0.05 was considered significant.

## Results

This was a retrospective analysis of 30 ICU patients with hypercalcemia (iCa ≥1.25 mmol/L). Out of the 30 patients, 15 were treated with pamidronate and 15 were not treated (controls). The mean age of the 30 patients was 54±13 years with an overall APACHE II of 22±9 and sequential organ failure assessment score (SOFA) score of 8±5 (Table 1).

**Table 1:** Baseline characteristics *mean±SD, †N (%), ‡mean (range) BMI=body mass index; APACHE=acute physiology and chronic health evaluation; SOFA=sequential organ failure assessment; SCr=serum creatinine; iCa=ionized calcium; IQR=interquartile range p<0.05 considered significant

Baseline characteristics	Control N = 15	Pamidronate N = 15	p-value
Age, years*	52 ± 15	56 ± 11	0.48
Gender, male†	14 (93)	11 (73)	0.33
Race†			
-Caucasian	3 (20)	2 (13)	0.99
-African American	9 (60)	12 (80)	0.99
BMI, kg/m^2^*	27 ± 6	23 ± 11	0.20
APACHE II*	21.6 ± 8.8	21.8 ± 10.4	0.95
SOFA*	7.0 ± 3.1	8.1 ± 5.7	0.53
SCr, mg/dL‡	1.5 (0.6 - 8.8)	1.2 (0.5 - 10.7)	0.45
Admission iCa, mmol/L*	1.12 ± 0.17	1.16 ± 0.27	0.66

The 15 hypercalcemic patients treated with pamidronate had an average ionized calcium (iCa) of 1.16±0.28 mmol/L; however, four of these patients were already hypercalcemic with iCa levels of 1.32, 1.40, 1.48, and 1.92 mmol/L. The other 11 patients who became hypercalcemic had an initial mean iCa of 1.03±0.17 mmol/L. The mean maximum iCa level was higher in pamidronate patients, (1.49±0.16 vs 1.33±0.07 mmol/L, p=0.002). The mean time from admission to treatment of hypercalcemia was 6.3±3.6 days with pamidronate compared to 18.2±12.2 days with controls, p=0.003. During that time, attempts were made to treat the hypercalcemia with dilution and diuretics.

Multiple laboratory studies were obtained just prior to the IV pamidronate (dose range 60-150 mg) and then daily for four more days. These laboratory studies included iCa, total serum calcium, sodium, potassium, chloride, carbon dioxide (CO2) content, serum creatinine, blood urea nitrogen (BUN), magnesium (Mg), phosphorus (Phos), and glucose (Gluc) (Table 2). The mean±SD initial lab values on day 1 prior to pamidronate administration included iCa 1.46±0.19 mmol/L, total calcium 10.3±2.6 mg/dL, albumin 2.2±0.6 gm/dL, albumin-corrected total calcium 12.0±0.8 mg/dL. Electrolyte values on day 1 included sodium 140±5 mmol/L, potassium 4.0±0.4 mmol/L, chloride 106±5 mmol/L, CO2 content 23.3±3.0 mmol/L, magnesium 1.9±0.3 mg/dL, and phosphorus 3.9±3.4 mg/dL. Other laboratory studies included BUN 34±23 mg/dL, serum creatinine 2.4±1.9 mg/dL, and glucose 110±21 mg/dL. 

**Table 2:** Laboratory data *mean±SD Na=sodium, K=potassium, Cl=chloride, CO2=dissociated bicarbonate, Mg=magnesium, Phos=phosphorus, BUN=blood urea nitrogen, Cr=creatinine, Gluc=glucose p<0.05 considered significant

Pamidronate patients	Reference range	Day 1	Day 5	p-value
Total calcium, mg/dL	8-10.6 mg/dL	10.3±2.6	7.9±0.3	0.001
Ionized Ca, mmol/L	1.13-1.32 mmol/L	1.46±0.19	1.15±0.10	<0.001
Na, mmol/L	135-147 mmol/L	140±5	140±4	0.99
K, mmol/L	3.5-5.3 mmol/L	4.0±0.9	4.1±0.6	0.72
Cl, mmol/L	96-112 mmol/L	106±5	109±3	0.05
CO2, mmol/L	20-30 mmol/L	23.3±3.0	20.4±4.9	0.06
Mg, mg/dL	1.6-3.0 mg/dL	1.9±0.3	2.1±0.3	0.07
Phos, mg/dL	2.3-5.0 mg/dL	3.9±1.4	4.4±1.8	0.40
BUN, mg/dL	7-20 mg/dL	34±23	50±40	0.19
Serum Cr, mg/dL	0.4-1.2 mg/dL	2.4±1.9	3.3±2.5	0.27
Gluc, mg/dL	70-99 mg/dL	110±21	116±34	0.56
Control patients		Day 1	Day 5	p-value
Total calcium, mg/dL	8-10.6 mg/dL	8.3±0.7	8.0±0.7	0.25
Ionized Ca, mmol/L	1.13-1.32 mmol/L	1.29±0.07	1.22±0.09	0.02
Na, mmol/L	135-147 mmol/L	140±5	140±4	0.99
K, meq/L	3.5-5.3 mmol/L	4.1±0.5	4.0±0.7	0.65
Cl, meq/L	96-112 mmol/L	107±5	105±6	0.32
CO2, meq/L	20-30 mmol/L	24.0±2.3	23.9±3.5	0.92
Mg, mg/dL	1.6-3.0 mg/dL	1.7±0.4	1.7±0.3	0.99
Phos, mg/dL	2.3-5.0 mg/dL	3.3±0.8	3.5±1.1	0.57
BUN, mg/dL	7-20 mg/dL	23±23	28±24	0.56
Serum Cr, mg/dL	0.4-1.2 mg/dL	1.5±2.1	2.0±3.2	0.61
Gluc, mg/dL	70-99 mg/dL	129±31	129±37	0.99

Following pamidronate, ionized calcium levels fell to < 1.25 mmol/L in two patients within 48 hours, in eight patients by 72 hours, and in 12 (80%) by 96 hours. One patient with severe renal dysfunction had an iCa of 1.92 mmol/L on day 1 which decreased to 1.32 mmol/L by day 5. This was successfully treated with a second dose of pamidronate. Two other patients with persistent hypercalcemia (iCa 1.25 and 1.27 mmol/L) four days after pamidronate responded to further dilution and diuretic therapy. Overall, the mean iCa declined after pamidronate from 1.46±0.14 to 1.15±0.01 mmol/L after four days which is a total reduction of 0.31±0.15 mmol/L for an average decrease in iCa level of about 0.08 mmol/L/day (Table 2). During the same time interval in the control group, the mean iCa only declined by 0.06±0.15 mmol/L (p<0.001). In addition, the total calcium decreased by 2.3±2.0 with pamidronate and only 0.3±1.1 mg/dL in the control group (p=0.002). In the control group, 7/15 remained hypercalcemic over the same time period even though the iCa levels in the control group were much lower (1.29±0.07 vs 1.46±0.14 mmol/L, p<0.001).

Following pamidronate therapy, sodium, potassium, and chloride levels were relatively constant; however, CO2 content tended to decline from 23.3±3.0 on day 1 to 20.4±4.9 mmol/L on day 5, (p=0.11), suggesting a slight tendency to metabolic acidosis. Phosphorus levels also increased slightly, from 3.9±1.4 on day 1 to 4.4±1.8 mg/dL on day 5 (p=0.01). The most definitive changes seen were in the BUN and SCr levels. The SCr increased from 2.4±1.9 on day 1 to 3.3±2.5 mg/dL on day 5 (p=0.001) and the BUN increased from 34±23 on day 1 to 50±40 mg/dL on day 5 (p=0.004). If the BUN and SCr significantly increased, there was a greater increase in the Cl 105±4 day 1 to 108±3 on day 4, mmol/L, and a decrease in the CO2 content 24.1±3.4 day 1 to 21.4±2.5 mmol/L on day 5. There were no significant laboratory differences between day 1 to day 5 in the control group (Table 2). In addition, hypercalcemic patients receiving pamidronate had a higher iCa and total serum calcium on day 1 compared to the control patients (Table 3).

**Table 3:** Impact on laboratory studies, control versus pamidronate *mean±SD Cl=chloride, CO2=dissociated bicarbonate, BUN=blood urea nitrogen, Cr=creatinine p<0.05 considered significant

Laboratory studies	Control N=15	Pamidronate N=15	p-value
Day 1*			
Ionized Ca, mmol/L	1.29±0.07	1.46±0.14	<0.001
Total calcium, mg/dL	8.3±0.7	10.3±2.6	0.007
Cl, meq/L	107±5	106±5	0.58
CO2, meq/L	24.0±2.3	23.3±3.0	0.47
BUN, mg/dL	23±23	34±23	0.20
Serum Cr, mg/dL	1.5±2.1	2.4±1.9	0.22
Day 5*			
Ionized Ca, mmol/L	1.22±0.0.9	1.15±0.10	0.05
Total calcium, mg/dL	8.0±0.7	7.9±0.3	0.61
Cl, meq/L	105±6	109±3	0.02
CO2, meq/L	23.9±3.5	20.4±4.9	0.03
BUN, mg/dL	28±24	50±40	0.07
Serum Cr, mg/dL	2.0±3.2	3.3±2.5	0.22

Parathyroid hormone levels (normal range 11-67 pg/ml) in the 15 pamidronate patients averaged 52±56 pg/ml. A chloride-to-phosphate ratio (Cl:Phos) of 33 or more is considered to be characteristic of hyperparathyroidism [11]. In the four patients with PTH levels of 60 pg/ml or more, the Cl:Phos ratio was 38±2. In the other 11 patients with a PTH of 45 or less, the Cl:Phos ratio averaged 30±9, (p=0.004).

In the 15 patients treated with pamidronate, only one of the eight with relatively normal renal function developed an increase in BUN or SCr after pamidronate. However, of the seven patients with moderate-severe renal dysfunction, four had further increases in SCr (5.4±1.5 to 6.0±0.4, mg/dL p=0.14) and five had a significant increase in BUN (59±22 to 81±38, mg/dL p=0.03). 

The seven patients with moderate to severe renal impairment had a slightly lower iCa than the eight patients with more normal renal function (1.42±0.24 vs 1.50±0.14 mmol/L, p=0.13). Comparing the eight patients with relatively normal renal function to the seven patients with impaired renal function, the mean iCa was identical on day 5 (1.15 mmol/L). 

Time from admission until the onset of hypercalcemia was shorter in the pamidronate group, (6±3 vs 18±12, days, p=0.003). As reflected by the increase in BUN and SCr levels, renal failure requiring dialysis tended to be more likely with pamidronate, 8/15 (53%) vs 3/15 (20%), p=0.12 (Table 4). Overall, 66% of all 30 patients developed an arrhythmia with the most common being sinus tachycardia (55%).

**Table 4:** Hypercalcemia variables and outcomes *mean ± SD, †N(%), ‡median (IQR) iCa=ionized calcium; LOS=length of stay; IQR=interquartile range p<0.05 considered significant

Hypercalcemia variables	Control N = 15	Pamidronate N = 15	P-value
Time from admit to hypercalcemia, days*	18.2 ± 12.2	6.3 ± 3.6	0.001
Hypercalcemia episodes‡	2 (1,2)	2 (1,3)	0.41
Peak iCa, mmol/dL*	1.33 ± 0.07	1.49 ± 0.16	0.001
Outcome			
In-hospital mortality†	2 (13)	3 (20)	0.99
Hospital LOS, days*	39 ± 32	51 ± 33	0.35
ICU LOS, days*	25 ± 22	41 ± 30	0.13
Mechanical ventilation duration, days* in 13 patients each	19 ± 21	28 ± 37	0.45
Renal dysfunction requiring hemodialysis†	3/15 (20)	8/15 (53)	0.06

The ICU length of stay (LOS) in the survivors tended to be longer in pamidronate patients, (41±30 versus 25±22, days, p=0.12). Hospital LOS was also longer in the pamidronate patients although not significant, 51±33 versus 39±32 days. In-hospital mortality was not significantly different, with 20% pamidronate versus 13% in controls (p=0.99) (Table 4).

Of the 30 patients, 11 (37%) had mild hypercalcemia (iCa 1.25-1.34 mmol/L), 11 (37%) had moderate (iCa 1.35-1.44 mmol/L), and eight had severe (iCa ≥1.45 mmol/L) hypercalcemia. There were no deaths in the 11 patients whose maximum iCa was 1.34 mmol/L or less. However, in the 19 patients with moderate to severe hypercalcemia (iCa 1.35 mmol/L or more), 5/19 (26%) died (p=0.12). Only two (18%) of the mild hypercalcemia patients required hemodialysis but it was needed in 9/19 (47%) of the moderate to severe hypercalcemic patients, (p=0.13). Severe hypercalcemia tended to be more likely in patients ≥ 60 years of age (5/8 (63%) vs 3/22 (14%), p=0.07).

Two of the patients who developed severe renal function within 48 hours of receiving pamidronate also received nephrotoxic drugs. These included colistin and vancomycin in one patient and vancomycin, aminoglycoside, colistin, and iodinated contrast in the other. Both patients required dialysis within seven days of the pamidronate therapy.

## Discussion

Hypercalcemia has been reported to occur in up to 23% of ICU patients. It reflects an increase in severity of illness, and ICU mortality rates tended to increase [1,3]. In the Egi study, mortality rates doubled if the iCa was > 1.25 mmol/L [1]. Indeed, mortality increased 2.6-fold with iCa levels > 1.35 mmol/L and almost tripled (OR 2.90) with iCa levels > 1.45 [1]. In addition, patients with the worst hypercalcemia tended to be male, older, and have a greater severity of illness [1].

Hypercalcemia can be life-threatening, especially when ionized calcium exceeds > 1.35 mmol/L [1]. Hypercalcemia can manifest as neuromuscular derangements (confusion, coma, muscle weakness), gastrointestinal problems (especially constipation), renal complications with inability to concentrate urine and eventual renal failure, cardiovascular changes (QTc interval shortening, arrhythmias), and skeletal alterations with increased fracture risk [2,4,8,12,13].

Generally, the treatment of hypercalcemia is aggressive hydration (dilution) with isotonic saline and diuretics. If hypercalcemia persists, especially if it becomes severe, bisphosphonates are increasingly being used. These agents are pyrophosphate analogues which lower calcium levels by inhibiting bone resorption by disrupting osteoclast activity. Pamidronate is also frequently used in the treatment of severe hypercalcemia from malignancies [2,8,13].

Cautions to using bisphosphonates include renal dysfunction, especially in patients with concomitant dehydration or potentially nephrotoxic agents [4,6,14]. Increases in serum creatinine have been reported in patients with osteolytic bone metastases up to 19% after pamidronate therapy. Pamidronate is known to cause focal segmental glomerulosclerosis with or without nephritic syndrome with drug information resources recommending single doses to not exceed 90 mg of pamidronate. In addition, adverse side effects of pamidronate also include fever, nausea, skeletal pain, and headache to name a few. After pamidronate therapy, asymptomatic hypophosphatemia, hypokalemia, hypomagnesemia, and hypocalcemia have been reported. We noted that the use of pamidronate in patients with moderate to severe renal dysfunction resulted in additional increases in the BUN and serum creatinine.

High bone turnover rates have been found in chronic severe illness through the disruption of osteoblastic bone formation, resulting in increased bone resorption. These high bone turnover rates have also been reported to increase all-cause mortality in elderly patients [15]. Pamidronate and etidronate have been studied for the treatment of hypercalcemia from immobility [2,6,16,17] which results in inhibiting bone resorption by disrupting osteoclast activity ultimately resulting in a decrease in serum calcium levels and adverse effects from hypercalcemia.

Schulman et al. studied the use of IV pamidronate in 30 out of 148 ICU patients with varying degrees of renal dysfunction [18]. None of the 30 patients who received pamidronate died while 22 (19%) of the controls died (p=0.007). Interestingly, no changes were noted in renal function in their patients after pamidronate. They also reported that pamidronate patients had a lower SCr on day 7 and day 9 [18].

Lucas et al. reported a 63% incidence of bradycardia at our institution in septic patients with multiple organ failure who also had hypercalcemia [7]. Hence, we expected to find a number of patients with life-threatening bradycardia, but it occurred in only 3/30 (10%) of the patients. Sinus tachycardia (14/30 (47%)) was the most common arrhythmia seen, especially at the higher iCa levels.

The main limiting factors in this study are the small sample size and the retrospective nature of this study. Based on previous literature, there may be a correlation with the parathyroid hormone function [4]; however, no clear correlation was found in our study.

## Conclusions

Hypercalcemia in ICU patients reflects increased severity of illness. In 80% of our patients, hypercalcemia resolved within four days of pamidronate therapy. It appears, however, that pamidronate may cause further renal dysfunction in patients with prior moderate to severe impairment. Prompt attention should be given to hypercalcemia in critically ill patients to minimize the possibility of life-threatening complications.
